# Optimal Magnetic Field for Crossing Super-Para-Magnetic Nanoparticles through the Brain Blood Barrier: A Computational Approach

**DOI:** 10.3390/bios6020025

**Published:** 2016-06-14

**Authors:** Maysam Z. Pedram, Amir Shamloo, Aria Alasty, Ebrahim Ghafar-Zadeh

**Affiliations:** 1 Department of Mechanical Engineering, Sharif University of Technology, Tehran, PO Box: 11365-11155, Iran; maysam.pedram@gmail.com (M.Z.P.); aalasti@sharif.edu (A.A.); 2 Department of Electrical Engineering, York University, Toronto, ON M3J1P3, Canada

**Keywords:** Molecular Dynamics simulation, force steering, Blood Brain Barrier, superparamagnetic nanoparticles

## Abstract

This paper scrutinizes the magnetic field effect to deliver the superparamagnetic nanoparticles (SPMNs) through the Blood Brain Barrier (BBB). Herein we study the interaction between the nanoparticle (NP) and BBB membrane using Molecular Dynamic (MD) techniques. The MD model is used to enhance our understanding of the dynamic behavior of SPMNs crossing the endothelial cells in the presence of a gradient magnetic field. Actuation of NPs under weak magnetic field offers the great advantage of a non-invasive drug delivery without the risk of causing injury to the brain. Furthermore, a weak magnetic portable stimulator can be developed using low complexity prototyping techniques. Based on MD simulation results in this paper, SPMNs can cross the cell membrane while experiencing very weak mechanical forces in the range of pN. This study also derives guidelines for the design of the SPMNs dedicated to crossing the BBB using external magnetic fields.

## 1. Introduction

**Figure 1:**
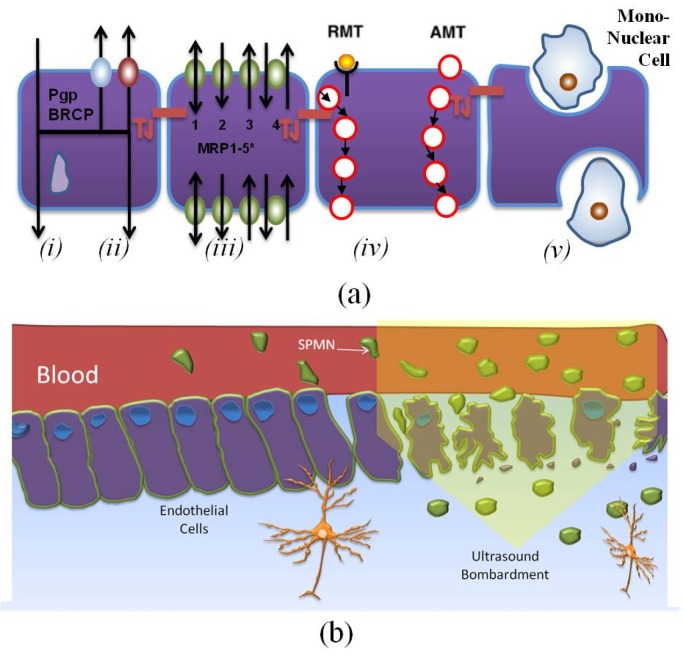
Methods of crossing the blood-brain barrier: (**a**) chemical methods and (**b**) a physical method (e.g., ultrasound). The chemical methods are separated into five different groups RMT (receptor-mediated transcytosis), AMT (adsorptive-mediated transcytosis), BRCP (bovine retinal capillary pericytes), and MRP (multidrug resistance protein). The physical methods rely on the effect of solid particles or electromagnetic/mechanical waves on endothelial cells.

Recent advances in micro- and nanotechnologies have greatly attracted the attentions of researchers for the development of magnetic nanoparticles (MNPs). MNPs offer great advantages for the diagnostics of various diseases, such as glioblastoma, Alzheimer, and Epilepsy [1,2,3]. MNPs have also demonstrated great advantages as contrast agents for Magnetic Resonance Imaging (MRI). Naturally occurring, the endothelium patterning around the cerebral micro vessels with strong tight junctions accurately controls the transportation of biomolecules necessary for neural signaling. However, the Blood-Brain Barrier (BBB) prevents the delivery of large drug molecules or NPs into the brain [4]. To overcome this problem, many papers have reported non-invasive chemical techniques to increase the permeability of the BBB [5,6,7]. Based on these papers, the improper surface chemistry hinders crossing through the BBB when the recognition of these NPs takes place along with receptors on the surface of the endothelial cells. Figure 1a shows the selectivity of these non-invasive chemical techniques that enable specific molecules to cross the BBB. As seen in Figure 1(a-i), there are many lipid-soluble molecules, or so-called *Passive partitioners*, that can cross the BBB and enter the Central Nervous System (CNS). The relation between the translocation rate and the degree of solubility of molecules in lipids has been elucidated in [5]. There are various methods for computing the permeability of the BBB for lipid-soluble non-polar molecules under the effect of passive diffusion. Diffusion parameters could then be evaluated based on experimental results. A rigorous formulation is provided for *in vivo* purposes. In addition to passive partitioners, there are many solutes and drugs called *ATP-Binding Cassette (ABC) transporters* that have much lower CNS entry rates than expected. These substances are actively transported through the capillary endothelium by means of the ABC transporter family (Figure 1(a-ii)). As mentioned above, the chemical techniques for crossing the BBB are selective and they allow the translocation of nutrients that are necessary for metabolisms, such as glucose and amino acids. Therefore, for transporting these essential nutrients to the brain, endothelium cells must contain a number of specific *Solute Carriers (SLCs)* which can play the role of transporters of those substances essential for brain activity and homeostasis. In addition, endothelial cells can use transport proteins for a wide variety of solutes and nutrients. Some of these transport proteins are polarized in their expression and are inserted into either the luminal or abluminal membrane only; others are inserted into both membranes of endothelial cells. The direction of the molecular transportation (e.g., Glucose, amino acids, nucleosides, monocarboxylates, small peptides, FFAs, organic anions, and organic cations ) is from the blood to the brain and *vice versa* (Figure 1(a-iii)).

The transportation of macromolecules through the BBB via endocytotic mechanisms is another important method for crossing the BBB. Although large molecular-weight solutes, such as proteins and peptides, are prevented from entering the CNS, there are a few transcytotic mechanisms capable of transporting large molecules. Figure 1(a-iv) illustrates such a mechanism. Transferin, melanotransferrin, lipoproteins, Amyloid β, clycosylated proteins, IgG, Insulin, Leptin, TNFα, and EGF (Epidermal Growth Factor) translocate to CNS via this mechanism. Furthermore, Figure 1(a-v) shows that cells from the bone-marrow derived monocyte lineage enter the brain during embryonic development and form colonies of residents and immunologically competent micro-glia. Moreover, mononuclear leukocytes, monocytes, and macrophages are able to be recruited by the CNS in pathological conditions, and play a complementary role to those of the resident micro-glia; in some cases they may transform into a microglial phenotype. Cationised albumin, histone, avidin, TAT, and synB are used in this method for facilitating the tansport.

Additionally, high-frequency electromagnetic field radiation and ultrasonic techniques have enabled the delivery of NPs or large biomolecules into the brain by creating nanoscale openings in the endothelium, as observed in Figure 1b [8,9,10,11,12,13,14,15,16]. Despite these advances, the development of a non-invasive or minimally invasive technique to deliver drugs or NPs into the brain is a key challenge. In this paper, we address this challenge by proposing a low complexity technique to deliver SPMNs in the brain.

**Figure 2:**
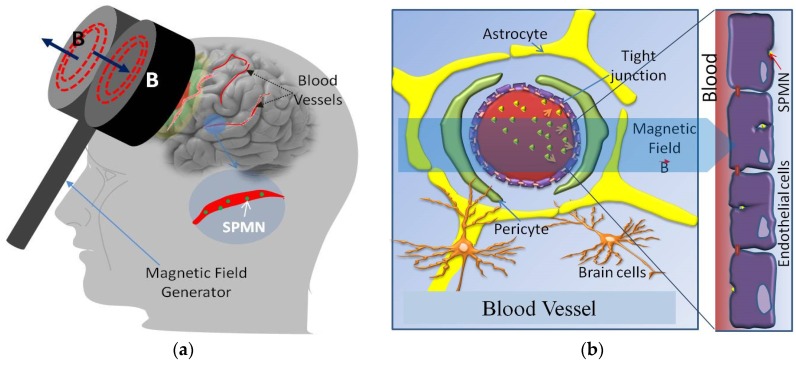
Magnetic superparamagnetic nanoparticles (SPMN) delivery into the brain: (**a**) Portable magnetic stimulator, and (**b**) Mechanism of crossing the blood brain barrier (BBB) based on the applied magnetic field. In this method, regarding the magnetic field applied to the magnetic nanoparticle, magnetic force created nanoparticles are crossing through the BBB.

As the result of this minimally invasive technique, these energized SPMNs penetrate through the membrane. Hence, we analyze the complexity of this technique using the molecular dynamic computational tool. This computational study deals with the molecular-level simulations to fathom the interaction between the energetic SPMNs and the endothelial cells membranes. Furthermore, this MD approach is crucial to obtain the optimal magnetic field energizing the SPMNs in order to cross the BBB. In other words, it is crucial to obtain the minimum gradient and/or amplitude of applied magnetic field for this purpose. Based on the literature, an MRI system can be practiced as a viable technique to energize such SPMNs [17,18]. As seen in Figure 2a, a handheld magnetic stimulator can also be used to generate the magnetic forces. Figure 2b reveals the structure of a capillary blood vessel. This figure also depicts how SPMNs translocate from the blood stream to the parenchymal tissue of the brain. An anisotropic transportation of SPMNs can be observed in this figure. In the remainder of this paper, the mathematical and MD models are described in Section 2 and Section 3. Section 4 demonstrates and discusses the results, and is followed by a conclusion in Section 5.

## 2. Mathematical Models

In this section, we present the simplified mathematical models of the required magnetic field for applying magnetic forces on SPMNs. In Section 4, we demonstrate and discuss the simulation results and practical guidelines using MATLAB and MD computational tools based on these models.

### Magnetic Force

The relationship between the magnetic force subjected on a magnetic dipole $\vec{m}$, in an applied magnetic field $\vec{B}$, is obtained from Equation (1):

(1) $$\vec{F}=\left(\vec{m}.\nabla \right)\vec{B}$$

By substituting $\vec{m}=\chi_{bead}V\vec{B}/\mu_{0}$ into Equation (1) [19], the following equation is obtained.

(2) $$\vec{F}=\frac{V\chi_{bead}}{\mu_{0}}\left(\vec{B}.\nabla \right)\vec{B}$$

where the magnetic susceptibility $\chi_{bead}$ and volume of the bead is expressed by $\chi_{bead}$ and *V*. $\mu_{0}$ is the permeability constant.

By assuming $\vec{B}=b_{z}{\vec{a}}_{z}$, the magnetic force is obtained from the following equation.

(3) $$\vec{F}=\frac{V\chi_{bead}}{\mu_{0}}\left(b_{z}\frac{\partial b_{z}}{\partial z}\right){\vec{a}}_{z}$$

By assuming that *b_z_* is a function of time and *z* or $Y\left(z,t\right)=b_{z}$ and $\vec{F}=F_{z}\left(t\right){\hat{e}}_{z}$, U(t) can be defined as follows.

(4) $$U\left(t\right)=\frac{F_{z}\left(t\right)\mu_{0}}{V\chi_{bead}}$$

*U*(*t*) is proportional to magnetic force *F_z_*(*t*), therefore, by substituting Equation (4) into Equation (3), and by assuming that Y(*z*,*t*) = *b_z_*, Equation (5) is achieved.

(5) $$Y\left(z,t\right)\frac{\partial Y\left(z,t\right)}{\partial z}=U\left(t\right)$$

Let us assume that the magnetic generator (e.g., MRI system) is linear ∂Y(z,t)/∂z=1/k where *k* is a constant value. Conclusively, the following equation is obtained.

(6) Y(z,t)=kU(t)

The magnetic field profile is obtained through extracting *U*(*t*) from the MD simulation results, based on Equation (6). The guidelines for designing the magnetic field will be discussed in Section 3.

## 3. Molecular Dynamic Models

In this section, we discuss the MD model of each component, including the BBB membrane and SPMNs. Then, we present the MD specifications.

### 3.1. MD Modeling

BBB Structure: The brain microvasculature is composed of three cellular elements including the BBB endothelial cells, astrocyte endfeet, and pericytes, as depicted in Figure 2. Tight junctions (TJs) present in the gap between the cerebral endothelial cells create a diffusive barrier, which selectively prevents the entrance of most blood-borne substances to the brain. The BBB endothelial cells differ from endothelial cells in the rest of the body by the absence of fenestrations, more extensively tight junctions (TJs), and sparse pinocytic vesicular transport [20]. In this study, only the cell membrane is modeled because the latter manifests no significant resistance to nano-particles when crossing the cytoplasm. It is noteworthy that this assumption is valid where the nanoparticle is crossing the cell membrane. In the case of crossing through tight junctions, a more complicated interaction should be modeled [21]. Selection of the correct size of NP would help to increase the rate of penetration. It has been experimentally observed that the tight junction can be opened for a NP with a size less than 20 nm, and this size of NPs can select this pathway for crossing the BBB [22]. A membrane is a lipid bilayer. In the literature, DMPC (DiMyristoyl PhosphoCholine) and POPC (Palmitoyl Oleyl Phosphatidyl Choline) are widely used to simulate BBB endothelial cell membranes. The membrane is a POPC lipid bilayer modeled by the VMD (Visual Molecular Dynamics) plugin Membrane Builder and merged by NP using a TCL (Tool Command Language) code [23,24].

**Figure 3:**
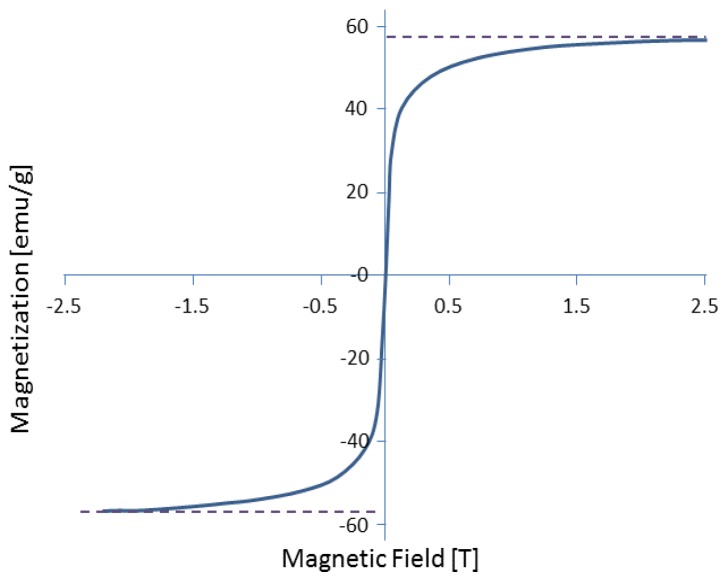
Magnetization curve for Fe_3_O_4_ magnetic suspension. In this figure, the threshold of saturation is demonstrated.

SPMNs Model: In this model, Iron Oxide (Fe_3_O_4_) is used as a core and gold (Au) is applied as a shell of the SPMNs. Au plays an important role in this approach. Au is a biocompatible material [25]. Moreover, Au has proven its advantage for crossing BBB in a non-invasive manner based on the experimental results reported in [22]. Additionally, the uniform distribution of Au in the brain after crossing the BBB has been also proved from the experimental point of view. However, the role of Au remains unknown in the above mechanisms [22]. Furthermore, it is also assumed that the magnetic saturation curve of the SPMNs is linear in this computational study [26]. Figure 3 illustrates the magnetization curve used in MD simulations. Based on this curve, $\chi_{bead}=0.17$ and the maximum magnetic field should be less than 2.5 T. In the literature, the rigidity of SPMNs is also argued [27] since the atomic bonds between the nano-particles of metallic atoms are much stronger than the chemical connections between (e.g., van der Walls interactions) the nano-particle atoms and the atoms of organic compounds are used in this study.

**Figure 4:**
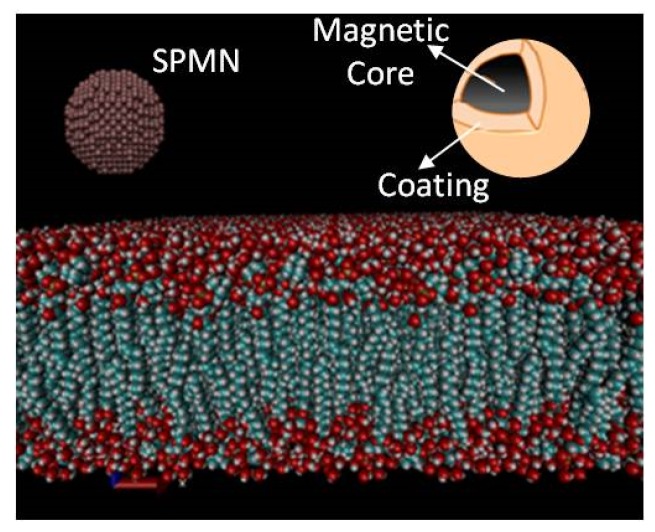
100 × 100 membrane and core/shell magnetic nanoparticle (NP).

Figure 3 shows the estimated strength of the magnetic field that can be applied on a NP. In regards to [28], coated and uncoated gold/magnetic NPs’ saturation values are similar, but coated NPs with gold saturation are a little higher, as reported in this paper. The maximum permitted magnetic field is defined, and it can be considered as the maximum value of the magnetic field. Although a higher magnetic field can be applied, due to the saturation effect, the effective magnetic force is the same. Figure 4 displays the 2 nm NP above the POPC membrane after 100 ps relaxation time. This figure indicates the structure of the NP. This NP forms a single rigid body with Au coating. Despite the fact that SPMNs can be considered as rigid particles, they are still deformable to some extent. Since Fe_3_O_4_ SPMNs are coated with Au, their structure can be considered as FFC (Face-Centered Cubic). In this MD modeling process, FCC is created using VMD plugin Inorganic builder. Then, a sphere with the desired radius is extracted from the FCC by a TCL code. Thus, the PDB (Protein Data Bank) and PSF (Protein Structure File) files were written. The van der Waals interactions are considered for all types of atoms (truncated at 12 A). This step runs for the second time using a Fe_3_O_4_ NP coated with gold. Thus, the material under the gold surface is considered in the interaction potential due to its cut-off distance [29]. It is noteworthy that in this computational study, the spheral-shape NPs are used. Therefore, the rotation of nanoparticles is not considered as a key factor. Due to the symmetrical shape of NPs, only the magnetic force in the defined direction is crucial. In [30], the direction of the magnetic field was taken into account; however, in this study, a magnetic field in a certain single direction is applied. It is noteworthy that, in the stability mode, the buoyancy force is canceled with the gravity force. The fluid resulting in the drag force is important, but in this study the NPs are located on the surface of enthotleial cells, and, in fact, we are supposed to calculate the force needed to cross through the BBB. The goal of this paper is to derive the minimum force required for crossing the BBB. Furthermore, it is well understood that Brownian motion is a function of temperature. In this study the Browning motion should also be taken into account. However, the average of Brownian motion is negligible due to the fact that the direction of the NPs’ motion is aligned with the direction of the magnetic field.

### 3.2. Molecular Dynamics Criteria

The MD simulation was performed with the Nanoscale Molecular Dynamics Package NAMD, and visualizations and analysis were performed using the Molecular Graphics Viewer VMD. The CHARMM27 All-Atoms (AA) force field was employed to model atomic interactions in the model. Based on the Langevin dynamics, all simulations were performed at a constant temperature equal to 310 K using a Langevin thermostat. In fact, the Langevin piston Nos’e–Hoover method was used to maintain a pressure at 101.3 kPa. The cutoff distance for the pairwise Lennard-Jones non-bonded interactions was set at 12 Å with a pair list distance extended to 14 Å.

Figure 4 reveals that the magnetic NP consists of two materia—Fe_3_O_4_ and gold. To date, many researchers, including León *et al*. [31], have reported the development of submicron hollow Fe_3_O_4_ that can be coated with Au; however, in this paper we want to put forward the manufacturing process not the magnetic properties of Fe_3_O_4_ coated with gold. The gold coating is needed for its biocompatibility, and also core-shell Au/Fe_3_O_4_ nanoparticles for drug delivery. As described in [32,33], the role of the gold shell functionalized with many functional groups is to increase the permeability of NPs crossing the BBB.

As already mentioned, all simulations consist of a cell membrane and a NP. The cell membrane is a POPC lipid bilayer with dimensions of 100 × 100 Å^2^, consisting of 50,318 atoms. Periodic boundary conditions are applied in all directions. The water molecules were modeled by the TIP3P method. Moreover, the time step is set to 2 fs. The initial energy of the system was minimized for 50 ps, thereafter the SMD (Steered Molecular Dynamics) simulation applied to the NP. This energy was used so that NPs cross the membrane at a constant velocity ranging from 0.0005 Å/Step to 0.1 Å/Step. During this process the exerted force on the nanoparticles was also recorded. The applied computational resources were necessary for the simulation. The simulation was performed on a personal workstation using a 2.67 GHz XEON processor for about seven days

## 4. Results and Discussion

In this paper, the MD modeling and simulation were carried out to investigate the non-invasive penetration of SPMNs into the brain through the BBB. Table 1 provides a summary of MD key parameters and results.

### 4.1. Magnetic Force

**Figure 5:**
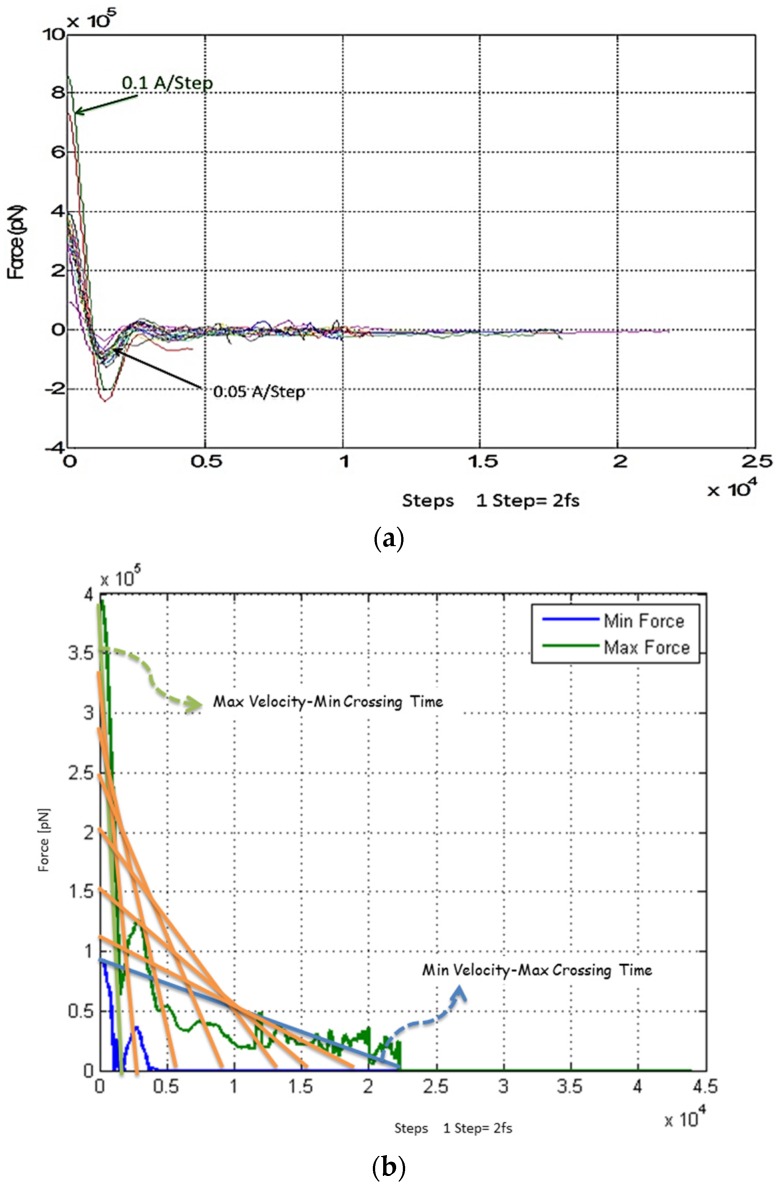

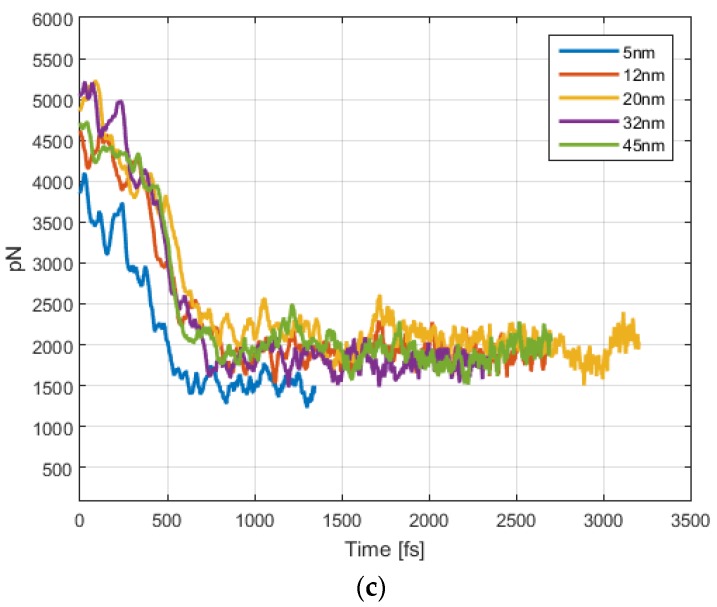
(**a**) Force Profile. Colored lines are related to different velocities of NPs. In this figure, the force profile needed for crossing nanoparticles is illustrated; (**b**) Diagram of minimum and maximum forces which are needed for crossing the BBB. Colored lines are related to different velocities of NPs; (**c**) Magnetic force *versus* time for different sizes of NPs ranging from 5 to 45 nm.

After 50 ps relaxation time, the NPs crossed the membrane at constant velocities ranging from 0.0005 Å/step to 0.1 Å/step. The applied force on the single NP as the effect of the magnetic field was recorded is shown in Figure 5a. As already discussed, a similar force profile must be applied on the NP in order to cross the BBB at the range of 0.0005–0.1 Å/Step. Based on our study, this figure shows that we need an alternative magnetic field in order to generate an appropriate force. As seen in Figure 5a, it needs a sharp burst of force amplitude. This mechanical force signal reaches its steady state after 3~4 oscillations. The rise time of this profile is about 10 pS (=2000 time steps). Figure 5a signifies that an effective SPMN translocation can be performed in such an oscillatory mode of acceleration for which we can discern the negative and positive cycles. Since a dummy spring has been considered as a means to express the softness of tissue in MD simulations, the oscillations are expected from the related governing equation of dynamic system. Despite this fact, moving in two directions could help crossing more easily, however, it results in a complicated magnetic field generator. Therefore, time and force are two factors that should be taken into account in order to push NPs effectively in a specific direction. Furthermore, the maximum amplitude of force when NPs pass the cell membrane plays a crucial role. Consequently, we omitted the negative oscillation and only the positive force in the effective direction was considered. For further simplicity, we can consider the minimum and maximum forces for crossing the BBB corresponding to two ranges of velocities. Figure 5b presents these minimum and maximum force values. It expresses that any force magnitude chosen from the interval between these two boundaries will assure crossing the BBB. In another simulation, different sizes of NPs have been examined. The applied forces on SPMNs with different sizes between 5 nm and 45 nm are obtained for crossing the BBB. Figure 5c shows that the SPMNs with various diameter sizes require different forces for crossing the membrane, however, these particles can move almost with the same force inside the cells.

### 4.2. Magnetic Field

In these MD simulations, a static magnetic field strength of 3[T] with a maximum spatial gradient magnetic field of 3.3[T/m] is singled out to achieve the required SPMN velocities. As already discussed, Figure 5a shows the magnetic force for energized SPMNs with different velocities. In other words, if the force applied on SPMNs is the same force applied to the membrane, the particles should move by a constant velocity. It is assumed that SPMNs can reach the assigned speed in this simulation in the presence of the aforementioned magnetic field’s strength and gradient. On the other hand, the magnetic field *Y*(*z*, *t*) can be obtained as shown and is discussed in Figure 5a based on the Equation (2). Since the maximum magnetic field value permitted for a nano-particle should not reach the saturation point, the effective magnetic field intensities simulated in this work should not have exceeded 2.5[T] for the core/shell (Fe_3_O_4_/Au). Based on the Equations (2) and (3), and using the constant values ($\mu_{0}$, V, $\chi_{bead}$) from Table 1, the applied magnetic field (*Y*(*z*,*t*)) should be 0.6 < *Y*(*z*,*t*) = $b_{z}$ < 2.5.

It is expected that the required time for crossing the membrane varies between 1.5 min to 7 min using various magnetic fields. The required time can be calculated through rescaling the travelling NPs in the upper velocity to lower velocities in the same distance and restricted magnetic field. Regarding these calculations, it is possible to exert alow magnetic field with higher duty cycle time and *vice versa*, while SPMNs can still cross the BBB. As described before, each endothelial cell features two barriers, one membrane when entering the endothelial cell and the other one when exiting the blood vessel. Inside the cells, the materials are softer than the membrane. As a result, the required force applied at a specific time is dedicated to pass the first barrier.

## 5. Non-Invasive BBB Crossing

**Figure 6:**
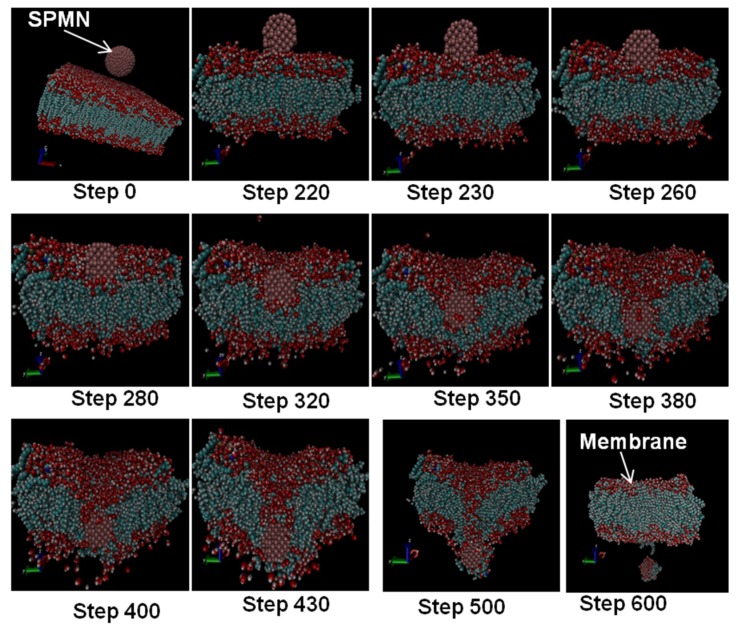
Several Steps of Crossing Through the BBB. These steps come from simulation, and the main goal is to show how the membrane is opened and how it can rehabilitate itself upon the completion of the crossing. In this figure, the receptor and insulin have been omitted in order to have a good view of the crossing.

**Figure 7:**
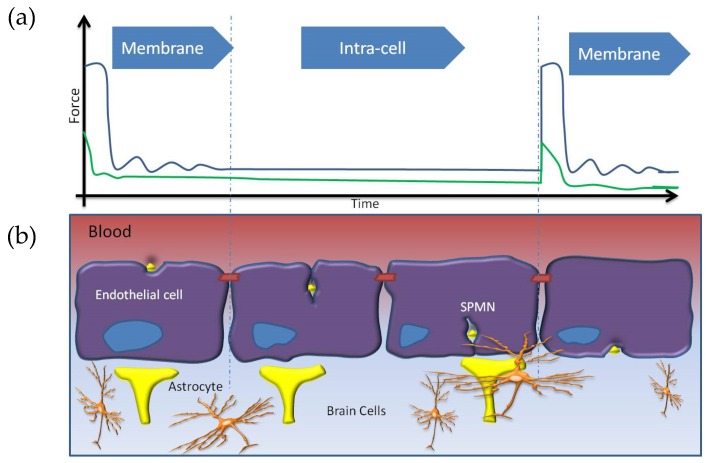
The process of NPs crossing through the BBB. The figure specifies how force should be exerted during the penetration of an endothelial cell and the penetration into the brain parenchyma. (**a**) force required for crossing through the cell vs Time. The blue line points to upper limit of the force and green line points to lower limit of the force. (**b**) status of nanoparticle inside the cell regarding the applied force during the time.

Figure 6 features the successive simulation frames at different moments for a few specific velocities. The steps in this figure were captured from the video output frequency. In this simulation, the video output frequency was a frame per 10 computational steps. It means that this figure was generated after 6000 steps of the numerical simulation when the nanoparticle crossed the BBB. As demonstrated in these images, the SPMN opens a gap in the membrane in order to cross the membrane. The corresponding deformation or other changes of the membrane are reversible. This process is totally non-invasive. Based on these simulation results, the SPMNs can cross the membrane by applying a magnetic force while these NPs can move through the cells with a much lower magnetic force, as shown in Figure 7.

## 6. Conclusions

In this work, we studied the advantage of using SPMNs in the presence of a gradient magnetic field to cross the BBB for disease diagnostics and subsequent treatment purposes. The core/shell (Fe_2_O_3_)Au structure with 2 nm diameter was used as a biocompatible SPMN. The focus of this paper was placed on the modeling and simulation of SPMNs in the course of crossing the endothelial membrane under a magnetic field. In this paper, we demonstrated new results showing the required magnetic field which can be obtained by simulating the applied force on SPMNs that interacted with the membrane molecules. Based on the simulation results, the magnetic field should be in the range of 0.6 T to 2.5 T. Also, depending on the nanoparticle size, the maximum magnetic force would be changed from 4000 pN to 5000 pN for 5 nm to 45 nm nanoparticle sizes, respectively. In addition, the crossing time is extracted according to the force profile diagram, and it can be controlled by varying the magnetic pattern and magnetic field strength during the process. The simulation results as depicted in Figure 5 and Figure 6 provide promising guidelines for the development of experimental protocols suitable for clinical applications.

## Figures and Tables

**Table 1:** Molecular dynamics key parameters.

Parameter	Quantity or Name
NP Type	$\left(Fe_{2}O_{3}\right)Au,\;\chi_{bead}=0.17$
NP Size	Diameter = 2 nm
Gold Thickness	2 A
Temperature	310 K, Langevin thermostat
Velocity of NPs	0.0005–0.1 Å /Step
Processor	XEON
Time	7 days
Relaxation time	20 nS
Membrane molecular dynamic (MD) model	POPC (Palmitoyl Oleyl Phosphatidyl Choline)
Membrane simulation area	*100 × 100 Å ^2^*
